# The Use of Deep Learning and Machine Learning on Longitudinal Electronic Health Records for the Early Detection and Prevention of Diseases: Scoping Review

**DOI:** 10.2196/48320

**Published:** 2024-08-20

**Authors:** Laura Swinckels, Frank C Bennis, Kirsten A Ziesemer, Janneke F M Scheerman, Harmen Bijwaard, Ander de Keijzer, Josef Jan Bruers

**Affiliations:** 1 Department of Oral Public Health Academic Centre for Dentistry Amsterdam (ACTA) University of Amsterdam and Vrije Universiteit Amsterdam Netherlands; 2 Department Oral Hygiene, Cluster Health, Sports and Welfare Inholland University of Applied Sciences Amsterdam Netherlands; 3 Medical Technology Research Group, Cluster Health, Sport and Welfare Inholland University of Applied Sciences Haarlem Netherlands; 4 Data Driven Smart Society Research Group Faculty of Engineering, Design & Computing Inholland University of Applied Sciences Alkmaar Netherlands; 5 Quantitative Data Analytics Group Department of Computer Science Vrije Universiteit Amsterdam Netherlands; 6 Department of Pediatrics Emma Neuroscience Group, Emma Children's Hospital Amsterdam UMC Amsterdam Netherlands; 7 Amsterdam Reproduction and Development Research Institute Amsterdam Netherlands; 8 Medical Library University Library Vrije Universiteit Amsterdam Netherlands; 9 Applied Responsible Artificial Intelligence Avans University of Applied Sciences Breda Netherlands; 10 Royal Dutch Dental Association (KNMT) Utrecht Netherlands

**Keywords:** artificial intelligence, big data, detection, electronic health records, machine learning, personalized health care, prediction, prevention

## Abstract

**Background:**

Electronic health records (EHRs) contain patients’ health information over time, including possible early indicators of disease. However, the increasing amount of data hinders clinicians from using them. There is accumulating evidence suggesting that machine learning (ML) and deep learning (DL) can assist clinicians in analyzing these large-scale EHRs, as algorithms thrive on high volumes of data. Although ML has become well developed, studies mainly focus on engineering but lack medical outcomes.

**Objective:**

This study aims for a scoping review of the evidence on how the use of ML on longitudinal EHRs can support the early detection and prevention of disease. The medical insights and clinical benefits that have been generated were investigated by reviewing applications in a variety of diseases.

**Methods:**

This study was conducted according to the PRISMA (Preferred Reporting Items for Systematic Reviews and Meta-Analyses) guidelines. A literature search was performed in 2022 in collaboration with a medical information specialist in the following databases: PubMed, Embase, Web of Science Core Collection (Clarivate Analytics), and IEEE Xplore Digital Library and computer science bibliography. Studies were eligible when longitudinal EHRs were used that aimed for the early detection of disease via ML in a prevention context. Studies with a technical focus or using imaging or hospital admission data were beyond the scope of this review. Study screening and selection and data extraction were performed independently by 2 researchers.

**Results:**

In total, 20 studies were included, mainly published between 2018 and 2022. They showed that a variety of diseases could be detected or predicted, particularly diabetes; kidney diseases; diseases of the circulatory system; and mental, behavioral, and neurodevelopmental disorders. Demographics, symptoms, procedures, laboratory test results, diagnoses, medications, and BMI were frequently used EHR data in basic recurrent neural network or long short-term memory techniques. By developing and comparing ML and DL models, medical insights such as a high diagnostic performance, an earlier detection, the most important predictors, and additional health indicators were obtained. A clinical benefit that has been evaluated positively was preliminary screening. If these models are applied in practice, patients might also benefit from personalized health care and prevention, with practical benefits such as workload reduction and policy insights.

**Conclusions:**

Longitudinal EHRs proved to be helpful for support in health care. Current ML models on EHRs can support the detection of diseases in terms of accuracy and offer preliminary screening benefits. Regarding the prevention of diseases, ML and specifically DL models can accurately predict or detect diseases earlier than current clinical diagnoses. Adding personally responsible factors allows targeted prevention interventions. While ML models based on textual EHRs are still in the developmental stage, they have high potential to support clinicians and the health care system and improve patient outcomes.

## Introduction

### Rationale

Digitizing meaningful health information has been proven to contribute to diagnostics. Electronic health records (EHRs) are a digital repository of patient data and contain retrospective, current, and prospective information supporting health care [1]. EHRs contain a wealth of clinical information about early symptoms of a disease and registries of medical treatments [2]. These can be textual or imaging data and include both unstructured clinical notes and structured, coded data. One important aspect of textual EHRs is that they may include risk and preventive factors and early signs before a disease manifests. Especially for patients with multiple visits, many possible indicators are gathered in EHRs, resulting in possible early indications of disease. Therefore, for a good risk assessment, clinicians need the patient’s health information, physical examinations, laboratory test results, and history [3] available in EHRs.

In the past 15 years, an explosion in the volume of data registered in EHR systems has occurred [4]. In 2012, the yearly increase in the volume of stored data was up to 150% for hospitals [5]. Not only the number of records continues to increase over time, but EHRs are also quite extensive because of large free texts [6]. Even though the completeness and correctness of EHRs have been found to be at a high level [7], the usability during medical visits lags behind due to this rising volume and variety of EHR data [8]. Consequently, it has even become an experienced usability issue for clinicians to review clinical results and health information from the past [9]. This is quite problematic as some clinicians spend, on average, 32.1% of their time on EHRs reviewing medical care and notes from the past [10]. The increasing EHR workload causes exhaustion and burnout among clinicians [11], negatively affecting the health care quality. This can result in diagnostic errors (missed, delayed, or incorrect diagnoses) because of missed signs [12] registered in the past. In 67.4% of the cases, missing the chief presenting symptoms in EHRs was the reason for missed diagnoses. Overall, meaningful health records have the potential to support risk assessment and early diagnosis, but the increasing amount of data hinder clinicians from using them to their full potential.

It is currently known that supportive tools can simplify complex diagnostic tasks and reduce potential diagnostic errors [13]. There is accumulating evidence suggesting that machine learning (ML) can assist clinicians in analyzing large-scale EHRs as they thrive on high volumes of data. ML is able to fit models specifically adapted to patterns in the data and, compared to traditional statistics, is able to handle multidimensional data [14]. Deep learning (DL) is a subdomain of ML that uses neural networks with multiple (hidden) layers, incorporating complex interactions between variables [15]. Examples of well-developed ML models are based on imaging data for disease detection [16,17] and textual EHRs of hospitalization or intensive care data for predicting disease progression or therapy success [18]. One of the most promising aspects of DL in the context of EHRs containing historical and present clinical data is the ability to incorporate temporality into the model, that is, to base possible risk assessments on hidden patterns over time in clinical parameters. Indeed, DL models have also proved to be more effective by incorporating temporal information (ie, longitudinally processed) rather than cross-sectional information only [19]. Although the techniques of many ML (including DL) models have proved to be effective on EHRs, their focus is often on the engineering of architectures and frameworks [20], but they lack medical outcomes.

### Objectives

It is a loss of information if ML developments remain unknown in health care because of the technical perspective of most authors. Especially given that artificial intelligence (AI) is a black box, it is important to clarify the clinical benefits and additional medical insights that can be achieved through these techniques. Therefore, the aim of this review was to perform a scoping review of the evidence on how the use of ML on longitudinal EHRs can support the early detection and prevention of diseases. A preliminary search was conducted, and no current or underway systematic or scoping reviews on the topic were identified. Only 1 review on longitudinal EHRs has been conducted [2], but it focused on methodologies. This study will contribute to what is already known by scoping the substantive medical insights that ML models yield. Given the aim of this study, the following research questions were addressed:

1. Which diseases have been detected in longitudinal EHRs using ML techniques?
2. What EHR data have been used by ML methods for the early detection and prevention of diseases?
3. What medical insights are generated by developing and using ML models on longitudinal EHRs?
4. What clinical benefits may be reached through the application of ML models on longitudinal EHRs?

## Methods

The conduct and reporting of this scoping review adhere to the PRISMA-ScR (Preferred Reporting Items for Systematic Reviews and Meta-Analyses extension for Scoping Reviews) statement [21]. A protocol has been registered in the Open Science Framework (DOI: NY2TE).

### Eligibility Criteria

Articles were included if they reported on early detection for timely prevention of diseases by using ML on longitudinal EHRs; the full description of eligible participants, concept, context, and types of sources can be found in the protocol. Overall, studies were screened according to several criteria.

#### Focus

Studies must have a clear focus on health care instead of a technical focus (eg, the article must include disease-specific information and interpretation, preferably executed and written from a health care perspective, and reflect on health or related care outcomes). Studies with a dominant technical focus or an engineering challenge or those using non–real-world data were assumed to be ineligible for this review.

#### Purpose

ML (including DL) should be aimed at predicting, detecting, or contributing to the risk assessment of diseases. Models aiming for data extraction, clustering, or patient selection for trials did not fit this concept. The purpose also affects the technique used.

#### Outcome

The prediction target of ML must be (the onset of) a disease or a medical event. By using the *International Classification of Diseases, 11th Revision* [22], we ensured that the primary outcomes were a disease or related medical event (ie, the cause of morbidity or mortality). Thus, studies that predicted disease severity once diagnosed, success of treatment, adverse drug reactions, phenotypes, or events that were not the cause of morbidity or mortality and did not focus on timely prevention were beyond the scope of this research. If the outcome was mortality, these articles were excluded because it is always a consequence of a disease or medical event.

#### Essential Elements of ML

Studies must incorporate the essential elements of ML, such as training, testing, or validation steps. DL was assumed as a subdomain within ML and, therefore, was included as well.

#### Data

According to the broadest definition of an EHR [1], data were assumed as EHR data if these contained information supporting continuing, efficient, and quality integrated health care or describing the health status of a patient regardless of the collecting database. Studies must use manually entered EHR data, including textual and numeric values. Both structured (numeric or coded) and unstructured (clinical notes) data were accepted as eligible EHR data. EHRs with solely imaging data (such as x-rays or electrocardiograms) were beyond the scope of this review. EHRs from animals were excluded.

#### Longitudinal

Studies must use EHRs over time registered at multiple visits (before registering a disease or medical event).

#### Context

Studies were included if they were conducted in the context of disease prevention. Optimal prevention in health care settings can be reached when participants at risk or signs of a disease are detected as early as possible, and therefore, these studies were eligible in the context of secondary prevention. Secondary prevention emphasizes early disease detection in subclinical forms and seeks to prevent the onset of illness [23]. Studies conducted using data gathered in intensive care settings during a hospital admission or data gathered at the emergency department cannot be viewed in the context of disease prevention because only tertiary preventive measures can be taken to reduce the effects or severity of the established disease as it is too late to influence the onset of disease.

#### Sample Size

Because ML is data driven (instead of conventional models that are hypothesis driven), only predictions based on >1000 participants in total were considered eligible. This threshold is based on theory (eg, calculations for multivariable predictions of binary outcomes [24]) and practice (eg, the range of sample sizes for disease prediction models on EHRs seen in the literature).

#### Study Design

Only study designs with clinical, real-world data were considered. If secondary research, such as other reviews, met the aforementioned criteria, the reference list was considered depending on the research question. Conference papers were also considered because of the high quality of evidence in computer science.

### Search

After several preliminary searches, 5 bibliographic databases (PubMed, Embase, Web of Science Core Collection [Clarivate Analytics], IEEE Xplore Digital Library, and computer science bibliography) were searched for relevant literature from inception to April 28, 2022. Searches were devised in collaboration with a medical information specialist (KAZ). The following search terms, including synonyms, closely related words, and keywords, were used as index terms or free-text words: “neural network,” “electronic medical record,” and “prediction.” We used only search terms capturing specific ML techniques that are able to predict or classify. The search strategy was adapted for each included database or information source. The searches contained no methodological search filter or date or language restrictions that would limit results to specific study designs, dates, or languages. We searched computer science bibliography for conference proceedings and hand searched meeting abstracts. Duplicate articles were excluded using the R package *ASYSD* (R Foundation for Statistical Computing), an automated deduplication tool [25], followed by manual deduplication in EndNote (version X20.0.3; Clarivate Analytics) by the medical information specialist (KAZ). The full search strategy used for each database is detailed in Multimedia Appendix 1.

### Study Selection

Following the search, all identified citations were collated and uploaded into Rayyan (Rayyan Systems Inc) [26] and EndNote (version X7.8). In total, 2 reviewers (LS and FCB) independently screened all potentially relevant titles and abstracts for eligibility. If necessary, the full-text article was checked against the eligibility criteria. Differences in judgment were resolved through a consensus procedure. The full texts of the selected articles were obtained for further review. As the aim was not to search for “the best available” evidence but to identify and perform a scoping review of all evidence, a critical appraisal was not systematically carried out.

### Data Extraction

Data were extracted from the papers included in the scoping review by 2 independent reviewers (LS and FCB) using a data extraction form developed in Microsoft Excel (Microsoft Corp). This form was composed based on full-text findings relevant to the research question and was discussed by the research team. The data extraction sheet captured details about study characteristics, health care discipline, generated medical insights, and clinical benefits for health care and the way EHRs were processed temporally. Multimedia Appendix 2 provides the list and definitions of all data items. This form was piloted using the first 5 articles and was revised and slightly adjusted during the process of extracting data. The extraction of ML techniques was modified to include the extraction of all techniques that were internally compared by appointing the central model and the comparison. Any disagreements between the reviewers were resolved through discussion with additional reviewers. Authors were contacted to request missing or additional data where required.

### Synthesis of Results

Extracted data were synthesized into results by frequency counts of concepts and qualitative narratives. Study characteristics, detected diseases, and EHR variables were listed in tabular form. The content of these tables was sorted by disease outcomes according to the *International Classification of Diseases, 11th Revision* disease categories from the World Health Organization. For data concerning medical insights and clinical benefits, a qualitative content analysis was carried out according to the guidance for scoping review knowledge syntheses [27,28]. After each study’s key findings were extracted, these were classified into concepts (1-6) and described using a narrative summary. We decided to describe both similarities and exceptions of the generated results and potential impact.

## Results

### Selection of Evidence

**Figure 1:**
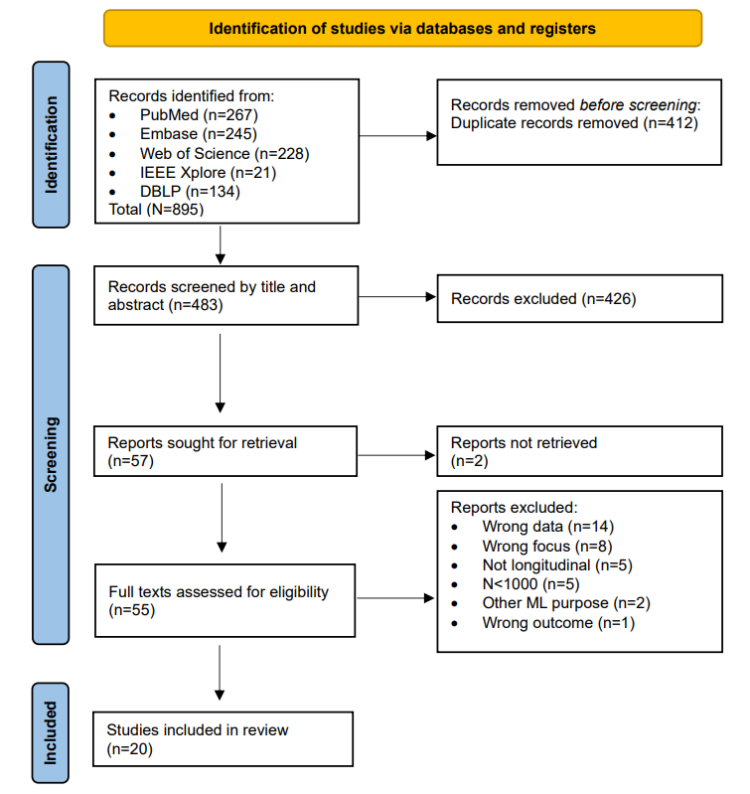
Flowchart of study selection. ML: machine learning. DBLP: DataBase systems and Logic Programming.

The literature search generated a total of 895 references. After removing duplicates of references that were selected from >1 database, 483 (54%) of the references remained. By screening titles and abstracts, 426 (88.2%) of the articles were excluded. Of the remaining 57 articles, 2 (4%) could not be retrieved because they contained unpublished work. In the second phase, 55 full texts were reviewed for eligibility, and ultimately, 20 (36%) articles were included. Reports were mostly excluded due to wrong data, a technical focus, the absence of a longitudinal aspect, or models based on N<1000. No additional studies were found by checking reference lists. After the final screening, most included articles (18/20, 90%) were found in PubMed. The flowchart of the search and selection process is presented in Figure 1.

### Characteristics of the Included Studies

Of the 20 included articles [29-48], 19 (95%) were published between 2018 and 2022, and 1 (5%) was published in 2016. The aim of these studies to develop an ML or DL model and examine whether it was able to detect the disease of interest in longitudinal EHRs. Detected diseases or related medical events were hepatocellular carcinoma [29], type 2 diabetes or prediabetes mellitus [30,31], mental health conditions [32], dementia [33,36], cognitive impairment [34], psychosis [35], heart failure [37], cardiac dysrhythmia [38], cardiovascular and cerebrovascular events [39], cardiovascular disease [40], knee osteoarthritis [41], kidney function decline [42,43], extreme preterm birth [44], opioid overdose [45], and suicide attempts [46]. One study proposed a health index [47] based on the prediction of 3 important health events, and another study predicted future disease in the next hospital visit [48]. Sample sizes ranged from thousands to millions. In total, 10% (2/20) of the studies used an external validation data set [35,39]. Table 1 shows the included studies and the detected diseases.

**Table 1:** Overview of the included studies and detected diseases.

Study, year		Disease or medical event	Aim of the study	Sample size, N
**Neoplasms**				
	Ioannou et al [29], 2020	Hepatocellular carcinoma	To examine whether deep learning recurrent neural network models that use raw longitudinal data extracted directly from EHRs^a^ outperform conventional regression models in predicting the risk of developing hepatocellular carcinoma	48,151
**Endocrine, nutritional, or metabolic diseases (diabetes)**				
	Alhassan et al [30], 2021	Prediabetes—HbA_1c_^b^ elevation	To identify patients without diabetes that are at a high risk of HbA_1c_ elevation	18,844
	Pimentel et al [31], 2018	Type 2 diabetes mellitus	To propose a new prognostic approach for type 2 diabetes mellitus given an EHR and without using the current invasive techniques that are related to the disease	9947
**Mental, behavioral, and neurodevelopmental disorders**				
	Dabek et al [32], 2022	Mental health conditions (anxiety, depression, and adjustment disorder)	To evaluate the utility of machine learning models and longitudinal EHR data to predict the likelihood of developing mental health conditions following the first diagnosis of mild traumatic brain injury	35,451
	Ford et al [33], 2019	Dementia	To detect existing dementia before any evidence that the GP^c^ had done so, that is, before they had started recording memory loss symptoms or initiating the process of dementia diagnosis	93,120
	Fouladvand et al [34], 2019	Mild cognitive impairment	To predict the progression from cognitively unimpaired to mild cognitive impairment and also analyze the potential for patient clustering using routinely collected EHR data	3265
	Raket et al [35], 2020	The first episode of psychosis	To develop and validate an innovative risk prediction model (DETECT^d^) to detect individuals at risk of developing a first episode of psychosis through EHRs that contain data from both primary and secondary care	102,030 (training)+43,690 (external validation)
	Shao et al [36], 2019	Dementia	To identify cases of undiagnosed dementia by developing and validating a weakly supervised machine learning approach that incorporated the analysis of both structured and unstructured EHR data	11,166
**Diseases of the circulatory system**				
	Choi et al [37], 2016	Heart failure	To explore whether the use of deep learning to model temporal relations among events in EHRs would improve model performance in predicting initial diagnosis of heart failure compared to conventional methods that ignore temporality	32,787
	Guo et al [38], 2021	Cardiac dysrhythmia	To predict cardiac dysrhythmias using EHR data for earlier diagnosis and treatment of the condition, thus improving overall cardiovascular outcomes	11,055
	Park et al [39], 2019	Cardiovascular and cerebrovascular events	To develop and compare machine learning models predicting high-risk vascular diseases for patients with hypertension so that they can manage their blood pressure based on their risk level	74,535 (training)+59,738 (validation)
	Zhao et al [40], 2019	Cardiovascular disease	To apply machine learning and deep learning models to 10-year cardiovascular event prediction by using longitudinal EHRs and genetic data	109,490
**Diseases of the musculoskeletal system or connective tissue**				
	Ningrum et al [41], 2021	Knee osteoarthritis	To develop a deep learning model (Deep-KOA^e^) that can predict the risk of knee osteoarthritis within the next year by using non–image-based electronic medical record data from the previous 3 years	1,201,058
**Diseases of the genitourinary system**				
	Chauhan et al [42], 2020	Rapid kidney function decline	To examine the ability of a prognostic test (KidneyIntelX) that uses machine learning algorithms to predict rapid kidney function decline and kidney outcomes in 2 discrete, high-risk patient populations: type 2 diabetes and APOL1-HR^f^	871 (data set 1); 498 (data set 2)
	Inaguma et al [43], 2020	Decline of kidney function (eGFR^g^)	To predict the rapid decline in kidney function among patients with chronic kidney disease by using a big hospital database and develop a machine learning–based model	118,584
**Conditions originating in the perinatal period**				
	Gao et al [44], 2019	Extreme preterm birth	To investigate the extent to which deep learning models that consider temporal relations documented in EHRs can predict extreme preterm birth	25,689
**External causes of morbidity (self-harm)**				
	Dong et al [45], 2021	Opioid overdose	To build a deep learning model that can predict patients at high risk of opioid overdose and identify the most relevant features	5,231,614
	Walsh et al [46], 2018	Suicide attempts	To evaluate machine learning applied to EHRs as a potential means of accurate large-scale risk detection and screening for suicide attempts in adolescents applicable to any clinical setting with an EHR	1470 (data set 1); 8033 (data set 2); 26,055 (data set 3)
**Multi-disease or other**				
	Hung et al [47], 2020	Health index	To propose a novel health index developed by using deep learning techniques with a large-scale population-based EHR	383,322 (training); 95,746 (testing 1); 102,625 (testing 2)
	Wang et al [48], 2020	Multi-disease	To explore how to predict future disease risks in the next hospital visit of a patient when discharged from a hospital	7105 (data set 1); 4170 (data set 2)

^a^EHR: electronic health record.

^b^HbA_1c_: glycated hemoglobin.

^c^GP: general practitioner.

^d^DETECT: Dynamic Electronic Health Record Detection.

^e^KOA: knee osteoarthritis.

^f^APOL1-HR: apolipoprotein L1 high-risk.

^g^eGFR: estimated glomerular filtration rate.

### EHR Data

The EHRs of patients used in the included studies were originally recorded in hospitals or primary care centers. Especially for the detection of mental and behavioral disorders, EHRs were often extracted from military health records [32,36], and for neurodevelopmental and cardiovascular disorders, EHRs were mostly extracted from general practices [33,37]. Most studies (16/20, 80%) used structured EHRs [29-33,35,38-43,45-48], sometimes combined with unstructured data [34,36,37,44], to estimate the risk of a disease or medical event. Demographic information (statically used), symptoms, laboratory (blood) test results, diagnoses, medications, BMI, and clinical notes were commonly used data from EHRs. In addition, the EHR length and hospital admission and visit history were frequently added to the model. Lifestyle data were included for cardiovascular diseases. Clinical and social signs were more frequently used for self-harm and mental, behavioral, and neurodevelopmental disorders. For the prediction of kidney and diabetes outcomes, laboratory test results were frequently extracted. If EHRs were unstructured, natural language processing methods were conducted as a precursor to analyze clinical notes. The central techniques were a basic recurrent neural network (RNN) or long short-term memory (LSTM) [29,31,34,35,39,44,45,49], often compared with logistic regression, support vector machine, or random forest. When techniques were used that could not handle temporal data, a temporal aspect was created in the data. Although not extensively specified, a slope and intercept of variables [31,36]; a mean [30]; minimum, maximum, median, and SD measures [42]; the addition of a time-weight (eg, 0.9 × days from reference point+decay) [43]; different time stamps [42]; or dividing the data into time blocks [33,46] were used. Multimedia Appendix 3 [29-48] provides an overview of the EHR data used and the techniques applied.

### Medical Insights

#### Overview

Disease detection and prevention can be supported by using ML or DL on longitudinal EHRs. First, the development and training of such models on EHRs can generate new medical insights (1-4). Second, when those models are applied (eg, for additional analyses or to “new” data in clinical practice), the following clinical benefits may be achieved (5 and 6). These insights will be summarized in the following sections.

#### Medical Insight 1: Diagnostic Performance

The use of ML and DL models on EHRs could support the detection of diseases with a high diagnostic accuracy. Performance metrics such as the area under the receiver operating characteristic curve (AUROC), sensitivity (recall), specificity, accuracy, precision, and the area under the precision-recall curve evaluated the detecting ability of the model. The AUROC was by far the most frequently reported metric because it illustrates the diagnostic ability for a binary classification (disease or nondisease) by using the sensitivity versus the specificity. Although it is not our intention to identify the best-performing model, it was observed that the AUROC of central models varied between 0.73 and 0.97. In 40% (8/20) of the studies, the optimal model had a “good” detection (AUROC between 0.7 and 0.8), 35% (7/20) of the studies succeeded in having a “very good” detection (AUROC between 0.8 and 0.9), and 15% (3/20) of the studies reached an “excellent” detecting performance (AUROC between 0.9 and 1.0) [36,41,46] according to the classification of diagnostic accuracy by Simundic [50]. For the best disease detection, multiple models were compared within the study, or the central model was compared with existing detection tools. The authors of 30% (6/20) of the studies claimed that their model produced a (slightly) higher performance than “conventional” or “traditional” models or ML models in the literature [29,34,37,38,44,45]. In 15% (3/20) of the studies, the central model performed better compared with currently used approaches such as a validated clinical model [42], a surveillance tool on which current health indexes are based [47], and a gold standard in routine clinical practice according to the American College of Cardiology and the American Heart Association [40]. In one study, the prediction scores of the model were validated by experts who agreed 100% through manual record reviewing [36]. The diagnostic accuracy of the included models was not dependent on disease categories but relied on the EHR data given to the model. Many studies (7/20, 35%) mentioned that diseases could be detected more accurately (ie, the predictive performance was increased) when the EHRs were closer to the date of diagnosis [32,33,46] and with an increase in the number of predictors [37,40,43,48]. Overall, the ability of the included models to classify nonhealthy and healthy individuals was close to the registered diagnoses in the EHRs.

#### Medical Insight 2: Earlier Detection

**Figure 2:**
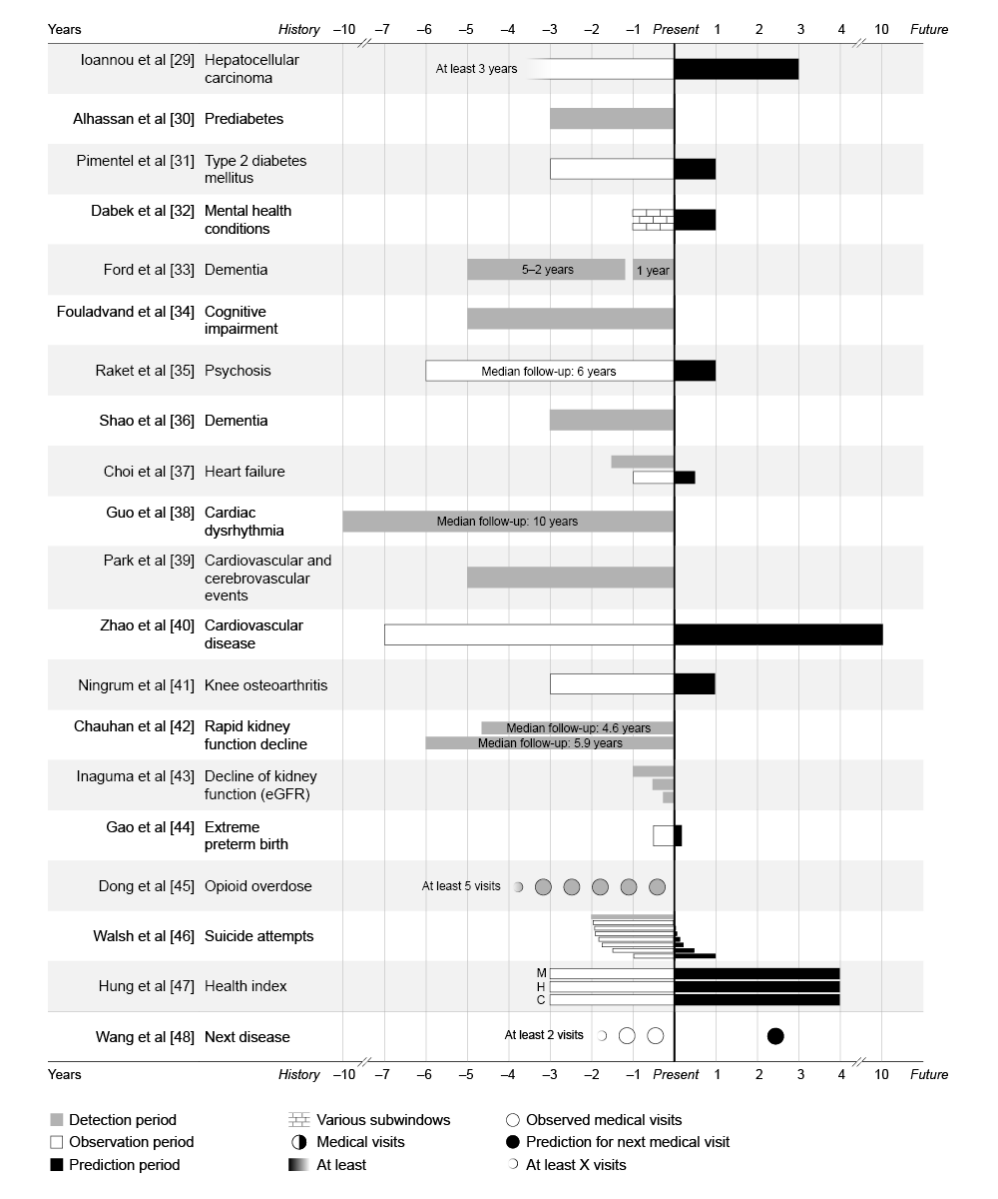
Detection, observation, and prediction periods per disease. A timeline of the electronic health record (EHR) periods that were used. The moment of the prediction (via machine learning) was scaled at x=0. Bars to the left (negative numbers) represent retrospective EHRs from years in the past, and bars to the right (positive values) represent predictions into the future. C: cancer; eGFR: estimated glomerular filtration rate; H: hospitalization; M: mortality.

In 45% (9/20) of the studies, ML and DL models observed all available EHR data to classify patients as a case or control (ie, ML vs human detection) [30,33,34,36,38,39,42,43,45]. However, in the other studies (10/20, 50%), models were able to detect diseases earlier than the moment they were diagnosed by clinicians in EHRs (ie, prediction) [29,31,32,35,37,40,41,44,46-48]. By dividing the participants’ EHRs into 2 pieces, X years were observed (observation period), and based on these data, it was possible to predict the risk of developing a disease or medical event in the future (prediction period). In other words, the prediction was made at an earlier time (x=0) than when it was diagnosed in practice (end of black bars). In some studies (5/20, 25%), it was part of the research to identify what time frame encompasses enough predictive information and, therefore, how much earlier an (accurate) detection was possible [32,33,37,43,46]. For example, Walsh et al [46] used 2 years of EHRs and extended their prediction window more and more to find the earliest moment of an accurate prediction. Raket et al [35] predicted whether a psychosis would occur 1 year before its onset, whereas Zhao et al [40] used 7 years of EHRs to predict the occurrence of cardiovascular events in the following 10 years. Figure 2 [29-48] illustrates the different time frames of longitudinal EHRs and their results according to a possible earlier detection. How much earlier a disease can be detected has a varying clinical meaning and, therefore, needs its own interpretation.

#### Medical Insight 3: Important Predictors

Another way to support disease detection and prevention was by generating insights into factors, topics, predictors, or indicators contributing to disease prediction [30,31,33,35-41,43-46]. In unstructured clinical notes, relevant topics, related words, and medical concepts were found that contributed to disease detection [36,44]. These words concerned daily living, behavior, and medical history. ML and DL models using structured EHRs generated the most contributing factors and their individual contribution to the outcome [30,31,33,35,37-41,43,45,46]. The most contributing predictors reported among all disease categories were (related to) age, blood pressure, BMI, cholesterol, smoking, and specific medication. Concerning mental, behavioral, and neurodevelopmental disorders, additional predictors were related to depression, personal difficulties, and personality changes. Some of these identified predictors were new for their discipline (eg, specific medication) [35,41,44] or not yet incorporated into gold standards for clinical diagnostic guidelines (eg, genetic information) [40]. In addition to this, insights into the importance of (known) predictors were generated. For example, Raket et al [35] identified what factors were responsible for the biggest positive and negative change in risk estimation (eg, differential white blood cells) and, therefore, indicated the most effective targets for preventive interventions. Other models found that the contribution of some predictors was not as high as assumed (eg, stress on diabetes) [31]; factors that seemed individually irrelevant turned out to have cumulative important predictive value [35], and the instability of factors, not the factor itself, was a predictor for one disease [40]. The aforementioned factors were identified during model development, but applying such a model to new EHRs would generate responsible factors for that individual.

#### Medical Insight 4: Other Health Care Indicators

In total, 10% (2/20) of the studies used EHRs not to predict the risk of a disease but to create other health indicators. Hung et al [47] developed a health index based on 3 DL predictions of impactful and costly health indicators (mortality, hospitalization, and cancer). This health index also generated insights into the population’s health and was found to be close to the “true risk” and, therefore, a better indicator than baseline models. Another study claimed to forecast what disease an individual would have at the next hospital visit [48]. Their results showed that the developed model generated well-performing results in forecasting medical diagnoses aggregated in 3- and 4-digit International Classification of Diseases, 9th and 10^th^ Revision codes.

### Clinical Benefits

#### Clinical Benefit 5: Preliminary Screening

In 25% (5/20) of the studies, ML models were used to support (preliminary) screening on longitudinal EHRs [29,35,36,42,46]. After developing ML and DL models, risk classes could be generated as a precursor for physical screening. Approximately 90% of the diagnosed cases were concentrated in the highest (10%) risk class. Other studies assessed the utility of ML and DL models by thresholds for the proportion needed to be screened versus the detection possibility [29,42]. For example, to detect 90% of all validated patients with hepatocellular carcinoma, the highest 66% of risk scores (predicted by a DL model) needed to be screened, whereas to detect 80% of all cases, screening from only the highest 51% of risk scores was required [29]. Chauhan et al [42] reasoned the other way around and focused on efficiency. From the 10% highest risk scores for kidney failure, the positive predictive value was 68%. Moreover, the cost benefits for screening options using DL on EHRs were investigated [35]. Disease detection using a DL model was associated with a positive net benefit–to–cost benefit ratio for a single-point risk assessment (1:3) and continuous-time risk assessment (1:16). Reasons for preliminary screening in EHRs were to prioritize those with the highest risk for disciplines with long waiting lists [29,42], before costly or more invasive examinations (eg, image or biomechanical retrieval) [35,41], or to detect cases that might be missed by the current pathway and go undetected [35,36,46].

#### Clinical Benefit 6: Possible Clinical Benefits

Only 10% (2/20) of the included studies were validated using an external data set, but none of the models have been implemented in clinical practice (yet). Consequently, the benefits for health were not evaluated. However, the authors interpreted their findings and suggested opportunities and possible health care benefits for clinical practice. The authors of 35% (7/20) of the studies mentioned that, if their models were applied in clinical practice, this may improve personalized health care [34-36,42,45-47]. Personalized health care was related to a personalized risk prediction, an individual-level index or output, a tailored care plan, and targeted care and screening. The authors of 60% (12/20) of the studies claimed that prevention could be improved by using their ML and DL models [31-38,42,44,45,47]. Early and timely detection and interventions before disease manifestation were often mentioned. In one case, the use of DL on EHRs could not directly prevent the targeted outcome, but by better preparing health care in an appropriate setting, indirect health outcomes could be prevented [44]. Additional suggestions to improve health care were focused on policies. It was suggested to base health policies on risk classes at a nationwide level [39,42]. Moreover, (predicted) future health conditions may be a better base for health care policies than traditional surveillance models reflecting health conditions from years before [47]. In addition to this, DL support can reduce the clinical workload. Even if the positive predictive value to select a screening population is low, a model with an excellent sensitivity can reduce the clinician’s workload by 70% [44]. All studies assumed EHR data to be valuable information to improve health care. The author of one study suggested that even imperfect data can be used as a silver standard to develop risk models [36].

## Discussion

### Summary of Evidence

The first research question in this study sought to determine which diseases have been detected in longitudinal EHRs using ML techniques. Results showed that a variety of diseases could be detected or predicted, particularly diabetes; kidney diseases; diseases of the circulatory system; and mental, behavioral, and neurodevelopmental disorders [22]. Comparing our findings with those of prior work, only a third of EHR prediction models predict diseases; meanwhile, mortality and hospitalization remain the most prevalent outcomes [51]. Among the studies that have predicted diseases, cancer is the most frequently predicted disease based on EHRs. Another systematic review used clinical notes to identify chronic diseases [52]. It also found diseases of the circulatory system as the most prevalent and explained this by the structure of the data. Not only the structure but also the length of the EHR horizon before diagnosis may explain the diseases that can be detected or predicted. As we determined the scope of diseases that may be prevented, the length of historic data before the diagnosis (in existence of early signs) reflects the “preventive stage” before the onset of the disease. The literature confirms that the longest EHR time horizon (8-10 years) has been found for diabetes and cardiovascular and kidney diseases [51], which were also prevalent diseases in our scoping review. In the end, the diseases that can be detected rely on available EHR data and, therefore, previous medical visits.

The second research question determined the scope of what EHR data have been used by ML techniques for the early detection and prevention of diseases. This scoping review found that age, sex, BMI, symptoms, procedures, laboratory test results, diagnoses, medications, and clinical notes are frequently used. Diseases that could be detected earlier than when they are currently diagnosed did not use other EHR variables. In addition, the most important predictors found in multiple studies were age, blood pressure, BMI, cholesterol, smoking, and medication. The consistency in the used and most important EHR variables underlines the importance of establishing generalized regulation and standardization of these variables across electronic health software, especially for variables overlapping in various health disciplines [53]. This would also address well-known challenges and limitations with EHR data, which will be discussed later in this section. According to the literature on the use of EHR data, it seems that a larger variable set improves disease prediction [51]. Their systematic review concluded that studies must leverage the full breadth of EHR data by using longitudinal data. In addition, we found that large longitudinal EHR data can successfully be analyzed via RNN and, derived from it, LSTM. These are both neural network architectures that are able to find patterns while incorporating temporality, making them effective for time-series predictions. Other types of neural networks (eg, convolutional neural networks) are well-known for their performance on images [15]. Similar results for techniques were identified in a review on the same topic from a technical perspective [2]. They concluded that RNN (specifically LSTM) was the most prominent technique to capture complex time-varying EHRs. Another review on AI techniques to facilitate earlier diagnoses of cancer also stated that neural networks were the dominant technique applied to EHRs [54]. Our results showed that there was no consistent way to process EHR variables temporally when techniques other than LSTM and RNN were used. Therefore, we can conclude that a basic RNN and LSTM are the most suitable techniques to analyze multivariable, longitudinal EHRs.

The third research question of this review was to determine the scope of medical insights that could be generated. Our results showed that, with the development and training of ML and DL models on EHRs, (1) a high diagnostic accuracy was reached, (2) the most responsible predictors could be identified, (3) diseases could be detected earlier than when they are currently diagnosed, and (4) additional health care indicators were created. The most prominent medical insight was the detection performance of the models. However, how good the performance should be is ambiguous. For example, DL models used to facilitate earlier cancer diagnoses had AUROC values ranging from 0.55 to 0.99 [54], indicating performance from almost random guessing to near-perfect detection. Looking into a more mature domain, the diagnostic accuracy of sepsis predictions ranged from between 0.68 and 0.99 in the intensive care unit to between 0.96 and 0.98 in hospital and between 0.87 and 0.97 in the emergency department [55]. This metric is ideally as high as possible because it induces a high sensitivity (true positives) and specificity (true negatives). For comparison, the diagnostic accuracy of a gut feeling (meta-analysis on cancer diagnosis) had a sensitivity of only 0.40 and a specificity of 0.85 [56]. The diagnostic accuracy of physical examination (for the detection of cirrhosis) had a sensitivity between 0.15 and 0.68 and a specificity between 0.75 and 0.98 [57]. If ML can increase both the sensitivity and specificity of disease detection, nonhealthy persons can be found, and delayed diagnoses can be reduced without overtreating healthy persons misdiagnosed as cases [58]. If the developed model is further evaluated in false-negative and false-positive groups, it may be possible that the model detects even more (true) cases than those registered by clinicians. This is already the case for many DL techniques on imaging data [59]. For now, an even more important finding is the ability of some models to detect disease manifestation earlier than the moment of diagnosis registration in EHRs. These examples of earlier detection are aligned with a study on the onset of diseases [60] that concluded that “slowly progressive diseases are often misperceived as relatively new” (ie, the onset could have been detected earlier). They found that, in 31% of diagnosed cases, the onset of their disease had started >1 year before their diagnosis. When disease predictions are early and accurate enough, it can facilitate disease prevention [23]. Especially with the addition of personally responsible factors and the biggest changers in risk prediction, prevention interventions may be more effective because they are more targeted to the individual. When medical prevention and interventions become based on the unique profile of each individual, personalized health care is delivered [61]. After all, the aforementioned medical insights only show the bright side of ML and DL models.

Our final research question sought the (possible) clinical benefits that could be obtained from using ML on EHRs. We found that preliminary screening was a clinical benefit of applying such models on longitudinal EHRs. Patients were accurately classified into risk classes to prioritize those with the highest risk, and a positive net benefit was found. In addition, the authors of the studies stated that their results (although they were not clinically evaluated) may contribute to a more personalized health care, prevention possibilities, and health care policies and reduce the clinicians’ workload. These benefits are perfectly aligned with the near-future vision, strategies, and action foci set by the World Health Organization [62,63]. In particular, the emerging clinical staff shortage makes the future health care system more dependent on technical innovations and the health care system will be forced to be digitally assisted [64]. However, to be adopted in medical practice, ML and DL models require external validation, the absence of bias and drift, and transparency for clinicians. In prior work, benefits have rarely been clinically evaluated either. Even in a more mature health domain regarding ML, the intensive care unit, only 2% of the AI applications are clinically evaluated [65]. In their systematic review, the clinical readiness of AI was explored, but no AI model was found to be integrated into routine clinical practice at the time of writing. The limited amount of publications evaluating the clinical benefits of the application of ML on EHRs indicates the research gap in the literature. Future studies should explore the follow-up of these AI attempts and the reasons for success or failure in practice.

Up until now, we have only discussed possible beneficial results of using ML and DL on EHRs. However, we cannot ignore the possible risks, obstacles, challenges, or issues. Multiple (systematic) reviews have summarized these well-known issues, challenges, and limitations regarding the application of ML and DL on EHRs [2,51,66,67]. Viewed generally across all studies, practical obstacles influence the scientific and clinical implementation process: ethical considerations, privacy guidelines, legal procedures, equity, and data protection and security [68]. Beyond these obstacles, existing predictions face limitations due to their reliance on the data. First, key issues of using EHRs are irregularity, heterogeneity, sparsity (eg, missing data), temporality, the lack of gold-standard labels, and the volume and quality of data [2,51,66,67]. Second, ML and DL models have limited transparency and interpretability, face domain complexity (vs engineering expertise), may include biases, and often lack external validation. It is not possible to assign specific issues to specific studies; they all suffer more or less from the aforementioned issues. Our point is to become aware of the downside as well. Therefore, all our principal findings must be interpreted with this last discussion point in mind. In our opinion, a consistent, reliable, and valid way of EHR registration will improve the (use of) data and could be the first step toward a data-based health care system. This need for movement and improvement is important not only for research but also for practical convenience for clinicians and, consequently, to succeed in improving health outcomes.

### Limitations

A limitation of this scoping review is the time between the search and the publication. As ML and DL have become a popular topic and the amount of research has grown drastically over the last years, new research could have been published between the literature search and the publishing of this scoping review. Consequently, some of our findings may have been overtaken by the progress in research.

Another limitation was the data synthesis regarding the performance outcomes per technique. Due to a wide variety of internal analyses, outcomes were not directly comparable, and therefore, the data extraction and data synthesis were difficult. Some studies just noted the optimal performance value achieved by the central model, while other studies compared a variety of techniques and noted various performance values for different subgroups, different metrics, and different time windows and with the addition of various technical improvements. A few authors discussed their ultimate results and mentioned that their model was better than literature, that is, “traditional” or “conventional,” attempts, which were not always clearly defined. We have attempted to follow the authors’ description to avoid incorrect comparisons. However, some comparisons may have become vague or skewed during data synthesis. Nevertheless, we scoped the optimal AUROC for each study at the meta level.

As we used a broad definition of EHR, we included a greater range of data. This means that the results are not based solely on data directly extracted from clinical record systems but also on data extracted by an intermediate organization, such as insurance companies. Therefore, readers must interpret the results of ML and DL models with this in mind.

### Conclusions

Longitudinal EHRs have valuable potential to support the early detection of a variety of diseases. For various diseases, EHR data concerning diagnoses, procedures, vital signs, medication, laboratory tests, BMI, and (early) symptoms have a high predictive value. To analyze multivariable, longitudinal EHRs, a basic RNN and LSTM are the most suitable techniques. For the detection of diseases, using ML (including DL) on EHRs proved to be highly accurate. When the detection occurs at the same moment as the diagnosis of clinicians, it seems not directly relevant for the prevention of diseases. However, the detection of diseases offers the clinical benefits of preliminary screening to prioritize patients from the highest risk class. The prevention of diseases can be supported by ML models that are able to predict or detect diseases earlier than the current clinical practice. The additional information about the most important predictors of the individual and the biggest risk changers allow targeted prevention interventions and, therefore, personalized care. Improved health care policies and workload reduction are frequently cited benefits but have not yet been evaluated in clinical practice. Both ML and DL attempts for disease detection and prevention still remain in the testing and prototyping phase and have a long way to go to be clinically applied.

## Appendix

Multimedia Appendix 1 Search strategy.

Multimedia Appendix 2 Data extraction instrument.

Multimedia Appendix 3 Electronic health record data and applied techniques.

Multimedia Appendix 4 PRISMA-ScR (Preferred Reporting Items for Systematic Reviews and Meta-Analyses extension for Scoping Reviews) checklist.

## Abbreviations

AI: artificial intelligence
AUROC: area under the receiver operating characteristic curve
DL: deep learning
EHR: electronic health record
LSTM: long short-term memory
ML: machine learning
PRISMA-ScR: Preferred Reporting Items for Systematic Reviews and Meta-Analyses extension for Scoping Reviews
RNN: recurrent neural network
