# Supplementary material for: The Use of Deep Learning and Machine Learning on Longitudinal Electronic Health Records for the Early Detection and Prevention of Diseases: Scoping Review
Source: J Med Internet Res. 2024 Aug 20;26:e48320. doi: 10.2196/48320 (PMC11372333; doi:10.2196/48320)
Supplement: Multimedia Appendix 1 [file jmir_v26i1e48320_app1.pdf]

Appendix 1: Search Strategy

PubMed Results (28 April 2022)

Search	PubMed Query – April 28, 2022	Results
#5	#1 AND #2 AND #3 AND #4	267
#4	temporal[tiab] OR longitudinal[tiab] OR time-series[tiab]	669,479
#3	predict*[tiab] OR detect*[tiab] OR risk*[tiab] OR diagnos*[tiab]	8,102,482
#2	"Electronic Health Records"[Mesh] OR electronic-health-record*[tiab] OR electronic-medical-record*[tiab] OR clinical-note*[tiab] OR medical-note*[tiab] OR computerized-patient-record*[tiab] OR hospital-information-system*[tiab]	62,411
#1	"Neural Networks, Computer"[Mesh] OR neural-network*[tiab] OR support-vector-machine*[tiab] OR Long-short-term-memory[tiab] OR text-min*[tiab] OR natural-language-process*[tiab] OR autoencoder[tiab] OR random-forest* OR deep-learn*[tiab] OR hierarchical-learn*[tiab]	135,342

Embase.com Results (28 April 2022)

Search	Embase Query – April 28, 2022	Results
#6	#5 NOT 'conference abstract'/it	245
#5	#1 AND #2 AND #3 AND #4	335
#4	temporal:ti,ab,kw OR longitudinal:ti,ab,kw OR 'time series':ti,ab,kw	852503
#3	predict*:ti,ab,kw OR detect*:ti,ab,kw OR risk*:ti,ab,kw OR diagnos*:ti,ab,kw	10919813
#2	'electronic health record'/exp OR 'electronic health record*' OR 'electronic medical record*' OR 'clinical note*' OR 'medical note*' OR 'computerized patient record*' OR 'hospital information system*'	140966
#1	'artificial neural network'/exp OR 'neural network*':ti,ab,kw OR 'support vector machine*':ti,ab,kw OR 'long short term memory':ti,ab,kw OR 'text min*':ti,ab,kw OR 'natural language process*':ti,ab,kw OR autoencoder:ti,ab,kw OR 'random forest*':ti,ab,kw OR 'deep learn*':ti,ab,kw OR 'hierarchical learn*':ti,ab,kw	166835

Clarivate Analytics/Web of Science Core Collection Results (28 April 2022)

Search	Web of Science Query – April 28, 2022	Results
#5	#4 AND #3 AND #2 AND #1	228
#4	TS=(temporal OR longitudinal OR time-series)	1,324,198
#3	TS=(predict* OR detect* OR risk* OR diagnos*)	11,606,719

#2	TS=(electronic-health-record* OR electronic-medical-record* OR clinical-note* OR medical-note* OR computerized-patient-record* OR hospital-information-system*)	49,190
#1	TS=(neural-network* OR support-vector-machine* OR Long-short-term-memory OR text-min* OR natural-language-process* OR autoencoder OR random-forest* OR deep-learn* OR hierarchical-learn*)	446,542

Institute of Electrical and Electronics Engineers [IEEE] Xplore Digital Library Results (28 April 2022)

**Search**	**IEEE Query – April 28, 2022**	**Results**
#1	(("neural network" OR "deep learning" OR "support vector machine") AND ("electronic health record" OR "electronic medical record" OR "computerized patient record") AND (prediction OR detection OR risk OR diagnosis) AND (temporal OR longitudinal OR "time series"))	21 (14 conference abstracts, 7 journals)

DBLP Computer Science Bibliography (14 juni 2022)

**Search**	**DBLP Query – June 14, 2022**	**Results**
**#1**	**(electronic health record|electronic medical record|computerized patient record)**	**134**

Appendix B: Data extraction instrument

Item	Definition
**Study characteristics**	
Author	First author of the study
Year of publication	The year in which the study was submitted.
Aim of the study	Objective from the introduction or abstract
**Healthcare**	
Database	The database from where EHR data were extracted
Origin of data	If available and different from database, the origin of the data, including the country.
Disease	The (diagnosed) disease of medical event related to a disease, that is targeted to predict by machine learning. Recognizable by an ICD-code.
**Healthcare knowledge**	
Key findings	Narrative description of the result section in the article. For example high/low precision, specific risk factors, importance of factors, prediction windows.
Clinical benefits	Narrative description of the (in)direct opportunities created by machine learning outcomes. What is the benefit to healthcare, f.e. related to clinical practice or policy. Indirect opportunities can also be found in the discussion section, where authors interpret results within their own domain and relate to their workflow at a higher level.
**Temporal machine learning**	
Type of data	Were EHR data structured, unstructured (clinical notes) or a combination?
EHR variables	The elements in EHRs that were used to develop the model. F.e. demographics, symptoms, comorbidity, lifestyle, history, examination findings or biometrics
Temporality	In what way were EHRs processed temporality? Via technique or data (manipulation/editing).
Machine learning techniques	All machine learning techniques that were used in the study. Machine learning models are often compared internally, to proceed with the best model to predict the disease. If concluded, the central or ultimate model was extracted as well. F.e. LR, SVM, RNN or LSTM.
External validation	Was the model validated in an external dataset? Yes/no

Sample size (trained:tested) and/or CV	Total number of included participants and the ratio of trained and tested data, f.e. 80:20. If a cross-validation was performed, the number of folds was extracted.
