# Supplementary material for: The Use of Deep Learning and Machine Learning on Longitudinal Electronic Health Records for the Early Detection and Prevention of Diseases: Scoping Review
Source: J Med Internet Res. 2024 Aug 20;26:e48320. doi: 10.2196/48320 (PMC11372333; doi:10.2196/48320)
Supplement: Multimedia Appendix 2 [file jmir_v26i1e48320_app2.pdf]

Appendix 2: Data extraction instrument

Item	Definition
**Study characteristics**	
Author	First author of the study
Year of publication	The year in which the study was submitted.
Aim of the study	The objective from the introduction or abstract
**Healthcare**	
Database	The database from where EHR data were extracted
Origin of data	If available and different from the database, the origin of the data, including the country.
Disease	The (diagnosed) disease of medical event related to a disease, that is targeted to predict by machine learning. Recognizable by an ICD-code.
**Healthcare knowledge**	
Key findings	Narrative description of the result section in the article. For example high/low precision, specific risk factors, importance of factors, or the prediction windows.
Clinical benefits	Narrative description of the (in)direct opportunities created by machine learning outcomes. What is the benefit to healthcare? F.e. related to clinical practice or policy. Indirect opportunities can also be found in the discussion section, where authors interpret results within their own domain and relate to their workflow at a higher level.
**Temporal machine learning**	
Type of data	Were EHR data structured, unstructured (clinical notes) or a combination?
EHR variables	The elements in EHRs that were used to develop the model. F.e. demographics, symptoms, comorbidity, lifestyle, history, examination findings or biometrics
Temporality	In what way were EHRs processed temporality? Via technique or data (manipulation/editing).
Machine learning techniques	All machine learning techniques that were used in the study. Machine learning models are often compared internally, to proceed with the best model to predict the disease. If concluded, the central or ultimate model was extracted as well. F.e. LR, SVM, RNN or LSTM.
External validation	Was the model validated in an external dataset? Yes/no

Sample size (trained:tested) and/or CV	Total number of included participants and the ratio of trained and tested data, f.e. 80:20. If a cross-validation was performed, the number of folds was extracted.
