# Supplementary material for: The Use of Deep Learning and Machine Learning on Longitudinal Electronic Health Records for the Early Detection and Prevention of Diseases: Scoping Review
Source: J Med Internet Res. 2024 Aug 20;26:e48320. doi: 10.2196/48320 (PMC11372333; doi:10.2196/48320)
Supplement: Multimedia Appendix 3 [file jmir_v26i1e48320_app3.pdf]

Table 2: Used EHR data and applied techniques

[a]MLR: Multiple Logistic Regression, [b]MLP: Multilayer Perceptron, [c]NB: Naïve Bayes, [d]ADT: Alternating Decision Tree, [e]RT: Random Tree, [f]KNN: k-nearest neighbors algorithm, [g]ANN: Artificial Neural Network, [h]LDA: Latent Dirichlet Allocation, [i]GRU: Gated Recurrent Unit, [j]DNN: Deep Neural Network, [k]DT: Decision Tree, [l]GB: Gradient Boosting (Tree), [m]CNN: Convolutional Neural Network.

	**Origin of data**	**EHR variables**								**ML and DL Techniques** (Central technique in bold)
		**Demographics**	**Lab results**	**Medication**	**Sympoms**	**Procedures**	**Diagnoses**	**Lifestyle**	**BMI**	
	**Neoplasms**									
[29]	Direct records from the Veterans Health Administration (VHA), United States.	✓	✓				✓		✓	Cross-sectional LR, longitudinal LR and **RNN.**
	**Endocrine, nutritional or metabolic diseases (diabetes)**									
[30]	Patient data taken from the King Abdulaziz Medical City, Saudi Arabia.	✓	✓						✓	MLR[a], RF, SVM, LR and **MLP[b]**
[31]	N.A. (diabetes database)	✓	✓	✓			✓	✓		**RF,** NB[c], SVM, **ADT[d],** RT[e], KNN[f] and MLP[b].
	**Mental, behavioral and neurodevelopmental disorders**									
[32]	Direct care records, collected in the Military Health System Data Repository (MDR), United States.	✓			✓					**Neural networks,** LR, RF, Adaboost and SVM.
[33]	Data from GP electronic patient record systems in primary care practices in the United Kingdom.			✓	✓					LR, SVM, RF, NB[c] and ANN[g].
[34]	N.A. (database Mayo Clinic Study on Aging)	✓			✓			✓		**1) NLP** **2) LSTM** and RF.
[35]	EHR data pooled from different healthcare systems with distinct EHRs.	✓	✓	✓		✓	✓		✓	**RNN**
[36]	EHRs from veterans who were previously evaluated at VA Puget Sound healthcare, United States			✓		✓	✓			**Topic modeling (LDA[h])→ LR.**
	**Diseases of the circulatory system**									
[37]	Data from a large primary care and multispecialty group practice in San Francisco, United States.	✓	✓	✓		✓	✓	✓		**1) NLP** **2) GRU[i],** LR, SVM, MLP[b] and KNN[f].

[38]	EHR data representing 70 clinics distributed throughout the United States.	✓		✓				✓		LSTM, **DNN[j],** RF, LR and NB[e].
[39]	Health records from all types of healthcare providers, covering the Korean population.	✓		✓			✓			LR, SVM, DT[k], **RF,** MLP[b], **LSTM.**
[40]	Routinely collected data from the insurance program in Taiwan.	✓	✓	✓			✓	✓		ACC/AHA equation, LR, RF, **GB[l], CNN[f]** and LSTM.
**Diseases of the musculoskeletal system or connective tissue**										
[41]	EHR data collected by the National Health Insurance Research and Development (NHIRD), covering 99,9% of Taiwan's inhabitants.	✓		✓			✓		.	**CNN[m]** + **ANN[g]**
**Diseases of the genitourinary system (kidney)**										
[42]	Patients' EHR from a diverse community in New York City, United States.	✓	✓	✓		✓	✓			**RF**
[43]	Electronic medical records of the Fujita Health University Hospital, Japan.	✓	✓							LR and RF
**Conditions originating in the perinatal period**										
[44]	EHR data of women who delivered newborns at VUMC.	✓	✓	✓		✓	✓			**1) NLP**<br>**2) LSTM,** LR, SVM and GB[l].
**External causes of morbidity (intentional self-harm)**										
[45]	Data from over 600 participating healthcare facilities.	✓	✓	✓	✓	✓	✓	✓		RF, ADT[d], LR, DNN[j], **LSTM +Attention**
[46]	Vanderbilt's clinical EHR data, including DNA.	✓		✓			✓			**RF** and LR
**Multi-diseases/other**										
[47]	Routinely collected data from the insurance program in Taiwan.	✓		✓			✓			**DNN[j]**
[48]	Health-related data about patients who stayed in the Beth Israel Deaconess Medical Center and a private dataset including inpatients with general care from a hospital in Shenzhen, China.	✓					✓			RNN, GRU[i] and **LSTM.**
